# Single-Needle Arthrocentesis with Upper Compartment Distension versus Conventional Two-Needle Arthrocentesis: Randomized Clinical Trial

**DOI:** 10.1155/2017/2435263

**Published:** 2017-10-03

**Authors:** Eduardo Grossmann, Primo Guilherme Vargas Pasqual, Rodrigo Lorenzi Poluha, Lilian Cristina Vessoni Iwaki, Liogi Iwaki Filho, Ênio Tadashi Setogutti

**Affiliations:** ^1^Craniofacial Pain Applied to Dentistry, Dentistry Faculty, Federal University of Rio Grande do Sul, Ramiro Barcelos Street 2492, 90035-004 Porto Alegre, RS, Brazil; ^2^Federal University of Rio Grande do Sul, Ramiro Barcelos Street 2492, 90035-004 Porto Alegre, RS, Brazil; ^3^Department of Dentistry, State University of Maringá, Mandacaru Avenue 1550, 87080-000 Maringá, PR, Brazil; ^4^Dental Radiology and Stomatology, Department of Dentistry, State University of Maringá, Mandacaru Avenue 1550, 87080-000 Maringá, PR, Brazil; ^5^Oral and Maxillofacial Surgery, Department of Dentistry, State University of Maringá, Mandacaru Avenue 1550, 87080-000 Maringá, PR, Brazil; ^6^Private Clinic, Freitas and Castro Street 481, 90040-401 Porto Alegre, RS, Brazil

## Abstract

The objective of this study was to compare single-needle arthrocentesis with distension of the upper compartment of the temporomandibular joint (TMJ) with the conventional two-needle arthrocentesis. Twenty-six patients with articular disc displacement without reduction (DDWOR) were included in the study and assigned to two groups (*n* = 13): single-needle arthrocentesis with distension of the upper compartment of the TMJ (1N) and conventional two-needle arthrocentesis (2N). The maximum interincisal distance (MID) and TMJ pain as measured by the visual analog scale (VAS) were compared. MID and VAS data were obtained: before (T1), seven days after (T2), fifteen days after (T3), one month after (T4), three months after (T5), six months after (T6), nine months after (T7), and one year after the arthrocentesis procedures (T8). Considering each group individually, results of the VAS scores and MID measurements showed a significant difference between T1 and T2–T8 (*p* < 0.001) in both groups. Between two groups, results show no significant differences (*p* > 0.05). Both techniques tested were effective in reducing pain and increasing MID. Due to the advantages over the conventional two-needle arthrocentesis, single-needle arthrocentesis with distension of the upper compartment should be considered as the first treatment option for patients with painful hypomobilized TMJ of DDWOR.

## 1. Introduction

Arthrocentesis is a minimally invasive surgical intervention of the temporomandibular joint (TMJ) [1, 2]. It is indicated for patients with joint disc displacement with and without reduction, disc adherences with mouth opening limitations, synovitis/capsulitis, painful articular noise during mouth opening/closing, and a palliative in acute cases of rheumatoid arthritis [3–6].

It consists of washing the upper compartment of the TMJ without the direct view of the TMJ, in which a biocompatible substance circulates with the purpose of diluting local pain mediators and relating substances to release the articular disc adherences formed between the disc surface with the articular eminence/mandibular fossa by the hydraulic pressure created by the irrigation [2, 5]. The conventional two-needle technique presents good results and low morbidity rates [6]. On the contrary the single-needle arthrocentesis and hydraulic distension of the upper TMJ compartment has been recommended by the relative simplicity of the technique, greater comfort for the patient, and shorter procedural time. With this technique, a needle is introduced under local anesthesia into the supradiscal compartment of the TMJ, followed by the injection, under pressure, of saline solution, anesthetic solution, Ringer's lactated solution, or sodium hyaluronate. Simultaneously, the patient is instructed to perform buccal opening movements and manipulate the mandible to reach maximum mouth opening [7–10].

In general, the literature suggests more investigations about the efficacy of different techniques. Thus, the aim of the present study is to compare the single-needle puncture arthrocentesis with the conventional two-needle technique. The null hypothesis to be tested is that there are no differences of efficacy between two techniques.

## 2. Materials and Methods

This research was approved by the Ethics and Research Committee on Human Beings of the Federal University of Rio Grande do Sul (CAAE: 60249716.0.0000.5347). All patients signed a free informed consent form, and a randomized clinical trial was conducted following the Helsinki Declaration and the recommendations of the Consolidated Standards of Reporting Trials [11].

A test power of 80% and significance level of 5% were considered for the sample size calculation. Adding 10%, for possible losses and refusals, the sample size was calculated at 26 individuals of patient. They were randomly divided into two groups: single-needle arthrocentesis with distension of the upper compartment of the TMJ (1N) and conventional two-needle arthrocentesis (2N) in number of 13 patients, respectively.

Inclusion criteria are patient/individuals should be over 18 years of age, with articular disc displacement without reduction (DDWOR) associated with unilateral joint pain complaint, who had not responded to previous conservative treatment (interocclusal device, anti-inflammatory drugs, light diet, and physical therapy) for at least three months who were selected for the study. Exclusion criteria are patients with rheumatoid arthritis, agenesis, hyperplasia, hypoplasia, and/or malignant neoplasm of the mandibular condyle, bone ankylosis, and previous TMJ surgery, with muscular disorders, and being allergic to any substances used in arthrocentesis, as well as individuals with extreme fear of needles.

The diagnosis was made by clinical examination, based on the Axis I of the Research Diagnostic Criteria for Temporomandibular Disorders [12] and confirmed by the findings of magnetic resonance images (MRI). All patients were treated between July 2015 and August 2016 at the orofacial pain and deformity center (CENDDOR) in Porto Alegre, Rio Grande do Sul, Brazil. All procedures were conducted by a single experienced author (EG). The following data were recorded from patients: gender; joint side with painful complaint; age (years); duration of joint pain (months); maximum interincisal distance (MID), measured in millimeters with a digital caliper (Mitutoyo®, Takatsu-ku, Kawasaki, Kanagawa, Japan); and pain perception (ranging from 0 to 10), measured with the visual analog scale (VAS). MID and VAS data were obtained in several times: before the arthrocentesis procedures (T1), seven days after (T2), fifteen days after (T3), one month after (T4), three months after (T5), six months after (T6), nine months after (T7), and one year after the arthrocentesis procedures (T8).

No additional infiltrations with any other substances were performed after the arthrocentesis. All patients received the same basic postprocedural care recommendations. Patients were blinded to the procedure and allocated to the groups according to a numeric draw performed in the immediate preoperative period.

### 2.1. Magnetic Resonance Images

MRI images were obtained in a 1.5 Tesla Signa HDxt equipment (GE Healthcare, Milwaukee, WI, USA). Series of T1 weighted images were performed with a time repetition (TR) of 567 milliseconds and time echo (TE) of 11.4 milliseconds. Series of T2 weighted images were performed with a TR of 5,200 milliseconds and a TE of 168.5 milliseconds, with a bilateral spherical surface coil of 9 cm in diameter. The matrix used for T1 was 288 × 192, with the number of excitation (NEX) = 3, while for T2 it was 288 × 160, with NEX = 4, and a field of view (FOV) of 11 × 11 cm.

In an attempt to minimize movement and keep maximum mouth opening, previously identified during clinical examination, the patient underwent MRI with an interoclusal device placed in the interincisal space. MRI images were all analyzed by the same radiologist, who based his analysis on the studies of Ahmad et al. (2009) [13].

### 2.2. Single-Needle Arthrocentesis with Hydraulic Distension of the Upper Compartment

The technique used was that recommended by the literature [7–9]. With the patients awake, they were asked to roll their head to the asymptomatic side. A straight line was drawn on the opposite side with a marker pen on the skin from the medial portion of the ear tragus to the lateral corner of the eye. In this line, a point for insertion of the needle was marked 10 mm from the middle point of tragus and 2 mm below the cantotragal line. After waiting for about 3 minutes for the ink to dry, antisepsis with a 2% chlorhexidine solution was conducted on the whole face, with emphasis on the preauricular region and ear. Afterwards, a sterile fenestrated surgical drape was placed on the face exposing the joint to be intervened, as well as a sterile ball of gauze next to the external acoustic meatus. The next step involved the anesthetic block of the auriculotemporal nerve with 2% lidocaine hydrochloride without norepinephrine 1 : 200.000 (tube of 1.8 mL), which was followed by the anesthesia of the posterior deep temporal and masseter nerves with one-two tubes. This resulted in excellent analgesia in the region, avoiding the need for sedation. The patient was asked to open the mouth as far as possible to allow the jaw head to move down and forward, facilitating the approach to the posterior recess of the upper TMJ compartment. A 40/12 needle (40 mm long and 12 mm thick) connected to a 5 mL syringe was inserted at the marked point. Approximately 4 mL of 0.9% saline solution (SS) was administered in order to distend the joint space. After the needle and syringe were removed, the patient was asked to perform opening and lateral movements of the mouth in order to break down any possible disc adherences, trying to restore an improved mandibular mobility pattern. Local dressing was conducted with sterile gauze and micropore.

### 2.3. Conventional Two-Needle Arthrocentesis

Conventional two-needle arthrocentesis was also performed according to the literature [1, 2]. On the intervention side, a straight line was drawn as described above. In this line, two points were marked for the insertion of the needles. The first was the same, while the second was 20 mm in front of middle point of tragus and 10 mm below on same line. The surgical area was prepared and the anesthetic blocks were performed as described above. With the patient with maximum mouth opening, a needle was introduced at the most posterior point, and 4 mL of 0.9% SS was administered. The second needle was introduced into the distended compartment, in front of the first needle, to initiate the lavage and joint lysis. A total of 300 mL of SS was used to perform TMJ arthrocentesis. Once the procedure was completed, the needles were removed and the patient was asked to perform the same mandibular movements described above. Local dressing was conducted with sterile gauze and micropore.

### 2.4. Statistical Analysis

Adopting the individual as the observational unit, the data were tabulated and subjected to descriptive analysis. In order to compare the variables of interest (VAS and MID) between interventions (1N and 2N) and evaluation times (T1, T2, T3, T4, T5, T6, T7, and T8), repeated measure ANOVA was used. All analyzes were performed with a significance level of 5% with SPSS, version 20.0, for Windows® (Microsoft Corporation).

## 3. Results

The 26 participating patients were evaluated over a period of one year. All patients returned to for all examinations (T1–T8). No complications during or after the procedures were reported. The frequency distribution (%) of gender, joint side of pain complaint, and the means and 95% confidence intervals (CI) of the variables age and pain duration are shown in Table 1.

Considering the within group differences, ANOVA results of the VAS scores showed a significant differences between T1 versus other times (T2–T8) (*p* < 0.001) in both groups. Between groups, considering the initial (T1) and other times (T2–T8), there were no significant differences (*p* > 0.05) (Table 2).

Considering the MID measurements, the within group difference was also found a significant difference between T1 and other times (T2–T8) (*p* < 0.001) in both groups. However in between group differences considering the MID data at initial (T1) and other times (T2–T8), there were no significant differences (*p* > 0.05) (Table 3).

## 4. Discussion

Arthrocentesis has been indicated as an effective treatment approach in patients with DDWOR [1, 5–7, 14]. Regarding the possible variations, the literature seems inconclusive when evaluating the different single puncture arthrocentesis techniques such as using two needles' fusion to one puncture devise [15] or using additional sodium hyaluronate with arthrocentesis [3]. In this randomized control study, the results supported the null hypothesis that there were no differences of efficacy between single and double puncture arthrocentesis in the variables VAS and MID.

In the present study, a significant reduction in VAS score was observed after the procedures, regardless of one or two-needle puncture technique for arthrocentesis (*p* < 0.001). This reduction in VAS score may suggest the removal of the inflammatory mediators during the arthrocentesis [16]. Another possible reason for the immediate reduction of the VAS score might be the anesthetic block of the auriculotemporal, posterior deep temporal, and masseter nerves. The literature suggests that adequate pain control during the procedure decreases painful stimuli to the central nervous system [4]. After one year of follow-up, the mean (±SD) VAS score reduced from 6.69 (±1.60) to 0.46 (±1.19) in group 1N and from 6.61 (±1.70) to 0.38 (±1.12) in group 2N. We should also consider the time factor in natural course of DDWOR. The evidences based reported data indicated that approximately 40% of patients with symptomatic DDWOR were free of symptoms within 2.5 years and one-third will improve, whereas one-quarter will continue to be symptomatic [17]. Whereas the present study of both single and double needle arthrocentesis showed significant reduction of pain immediately and to long run as a useful treatment interventions.

By the arthrocentesis, there was an increase in MID in all our patients (*p* < 0.001) in both groups, but with no significant differences (*p* > 0.05) in between two groups. The beneficial effects of arthrocentesis on mandibular joint mobility are likely due to the enlargement of the joint space, increased intra-articular pressure, removal of adhesions and adherences, and alterations to the synovial fluid viscosity [1, 10, 18–20].

Some advantages have been attributed to single-needle arthrocentesis. It can reduce procedural time and limit trauma [10, 15, 21]. Single-needle arthrocentesis with hydraulic distension of the upper compartment is particularly useful in cases of joint hypomobility or with adherences. Because of the confinement and restriction of the injected solution, the intra-articular pressure may be higher than that obtained with conventional two-needle arthrocentesis and may work favourably to release of the disc [7, 10].

Lastly the caution should be excessed that this is a monocentric study with a restricted population. New studies replicating the same design should be considered to evaluate the surgical comfort experienced by patients during the procedure. Analysis of the synovial fluid and postoperative MRI evaluation would also be important to understand tissue response to different arthrocentesis techniques, which could be used to predict the outcome of the procedure.

## 5. Conclusions

Considering the limitations of the present study, the results obtained indicate that both tested arthrocentesis techniques were equally effective in reducing the pain and increasing MID in patients with DDWOR. Due to the advantages over the conventional two-needle arthrocentesis, single-needle arthrocentesis with distension of the upper compartment should be considered as the first treatment option for patients with painful hypomobilized TMJ of DDWOR.

## Figures and Tables

**Table 1:** Frequency distribution, means, and confidence intervals of the descriptive variables of the sample.

Variables	Group 1N (*n* = 13)	Group 2N (*n* = 13)
Gender		
Female	9 (69.30%)	13 (100%)
Male	4 (30.70%)	0 (0.00%)
Side of the complaint		
Right	6 (46.15%)	5 (38.46%)
Left	7 (53.85%)	8 (61.54%)
Age (years)	42.1 (32.8–51.5)	39 (29.5–48.4)
Pain duration (months)	69.5 (14.9–124.1)	51.2 (11.3–91.1)

**Table 2:** Comparison of visual analog scale (VAS) scores (0–10) according to the follow-up time between groups 1N and 2N.

Times	Group 1N Means (±SD)	Group 2N Means (±SD)	*p* value
T1	6.69 (±1.60)	6.61 (±1.70)	0.821
T2	1.15 (±1.86)	1.15 (±1.77)	0.796
T3	0.92 (±1.44)	0.69 (±1.54)	0.863
T4	0.84 (±1.90)	0.61 (±1.70)	0.797
T5	0.61 (±1.55)	0.53 (±1.33)	0.745
T6	0.46 (±1.19)	0.38 (±1.12)	0.711
T7	0.46 (±1.19)	0.38 (±1.12)	0.711
T8	0.46 (±1.19)	0.38 (±1.12)	0.711

T1: before the arthrocentesis procedures; T2: seven days after the arthrocentesis procedures; T3: fifteen days after the arthrocentesis procedures; T4: one month after the arthrocentesis procedures; T5: three months after the arthrocentesis procedures; T6: six months after the arthrocentesis procedures; T7: nine months after the arthrocentesis procedures; T8: and one year after the arthrocentesis procedures. SD: standard deviation.

**Table 3:** Comparison of maximum interincisal distance (MID) (mm) according to the follow-up time between groups 1N and 2N.

Times	Group 1N Means (±SD)	Group 2N Means (±SD)	*p* value
T1	31.82 (±2.51)	32.83 (±4.70)	0.497
T2	43.83 (±4.28)	45.19 (±5.06)	0.527
T3	43.51 (±4.42)	45.13 (±5.03)	0.590
T4	43.38 (±4.49)	44.94 (±4.92)	0.757
T5	43.29 (±4.47)	45.03 (±4.94)	0.672
T6	43.16 (±4.44)	44.99 (±4.98)	0.670
T7	43.02 (±4.43)	44.76 (±5.10)	0.584
T8	42.94 (±4.45)	44.69 (±5.36)	0.585

T1: before the arthrocentesis procedures; T2: seven days after the arthrocentesis procedures; T3: fifteen days after the arthrocentesis procedures; T4: one month after the arthrocentesis procedures; T5: three months after the arthrocentesis procedures; T6: six months after the arthrocentesis procedures; T7: nine months after the arthrocentesis procedures; T8: and one year after the arthrocentesis procedures. SD: standard deviation.
